# Impact of Virtual Education Versus Traditional Learning Modalities on the Body Mass Index of Students During the COVID-19 Pandemic in Western Saudi Arabia: A Cross-Sectional Study

**DOI:** 10.7759/cureus.22596

**Published:** 2022-02-25

**Authors:** Abdulmoein Al-Agha, Rafeef Alsalmi, Bodoor Alahmadi, Teif Aljilani, Sarah Alasbli, Fai Alsharef, Ghaidaa Baothman, Janan Maddah

**Affiliations:** 1 Pediatrics, King Abdulaziz University Hospital, Jeddah, SAU; 2 Medicine, King Abdulaziz University, Jeddah, SAU

**Keywords:** children, body mass index, education, traditional, virtual

## Abstract

Objective: The emergence of the coronavirus disease has forced governments worldwide to implement non-pharmaceutical interventions that imposed strict confinement policies on their populations, including children and adolescents. Subsequently, the education system has shifted from traditional to online classroom settings, introducing physical and lifestyle changes in students. This study aimed to determine the impact of virtual education in comparison with that of traditional education on body weight among school-age students in western Saudi Arabia.

Methods: This cross-sectional study was carried out between March 2020 and May 2021 and assessed pediatric patients at the Pediatric Endocrine Clinic at King Abdulaziz Hospital, Jeddah, Saudi Arabia. A clinical interview was performed to collect data on sociodemographic characteristics, physical activity levels, and electronic device usage. Body mass index (BMI) was determined using the Center for Disease Control and Prevention standards.

Results: In total, 518 participants (262 female and 256 male students) aged six to 18 years were assessed. The BMI significantly increased from a mean of 19.24, before the pandemic, to 20.08, after the commencement of the virtual study modality (p<0.001) that involved the increased use of electronic devices. Moreover, the proportion of physical inactivity significantly increased during weekdays (39%) and weekends (37.5%).

Conclusion: Due to the many obesogenic factors influenced by the coronavirus disease 2019 (COVID-19) pandemic, the findings indicate the need for further research and interventions to avoid the incidence of overweight and obesity among students. Many obesogenic factors are influenced by the COVID-19 pandemic, which affect the student's physical activity and lifestyle by increasing the risk of overweight and obesity. Therefore Further research and interventions are needed.

## Introduction

The emergence of the coronavirus disease 2019 (COVID-19) has forced governments worldwide to implement non-pharmaceutical interventions, such as social distancing, which imposed strict confinement policies on their populations including children and adolescents. Since March 2020, all Saudi citizens have been urged to limit their social contact and live in seclusion for several months because of the COVID-19 pandemic. As a result, the education system has witnessed an abrupt shift from the traditional classroom setting to an online classroom setting in accordance with social distancing and stay-at-home directives. All of these policies have had socioeconomic, psychological, and health consequences [1].

Subsequently, school closures and online classes during the COVID-19 pandemic may exacerbate obesity because students have fewer opportunities for group exercise due to the lack of physical education programs and school activities. Furthermore, the imbalance between calorie intake and physical activity causes weight gain, which is considered a long-term effect of the pandemic on students. Globally, obesity is a significant public health concern that is caused by interactions between hereditary and environmental variables [2-4]. Of note, there are few studies that have shown how childhood and adolescent obesity and physical activity patterns have evolved during the COVID-19 pandemic. One of the many recent studies that focused on this pandemic showed that screen time was correlated with obesity among students. Moreover, a study in an American population found that an increased risk of diabetes, obesity, and cardiovascular diseases would be potentially attributable to a lower level of physical activity and increased screen-time (hours) if the changes in response to the COVID-19 pandemic were to become permanently entrenched [5]. Therefore, this study aimed to determine the impact of virtual education with lack of physical activity during the COVID-19 pandemic on body weight in comparison with the effect of the traditional education-delivery setting among school-age students in Jeddah, Saudi Arabia.

## Materials and methods

Participants and instruments

Between March 2020 and May 2021, we conducted a cross-sectional study at the Pediatric Endocrine Clinic at King Abdulaziz Hospital, Jeddah, Saudi Arabia. The participants were 518 students (age: six to 18 years; 49.5% males, 50.4% females). We collected the study-related information in three phases, specified by a four-month interval - phase 1, the first term from September 2019 to January 2020 (pre-COVID-19); phase 2, the second term from January 2020 to May 2020; and phase 3, both terms from September 2020 to May 2021. Height and weight data were obtained from hospital records. We recorded the participants’ weight to the nearest 0.1 kg using a single scale, and their height (without shoes) to the nearest centimeter using a mechanical beam scale with a height rod.

Data collection

The body mass index (BMI) is used to screen for weight-related health issues. We calculated the BMI as weight (kg) divided by height (m^2^), and classified the students as either normal (5th-85th percentile), overweight (85th-95th percentile), obese (>95th percentile), or severely obese (>99th percentile) using the age-stratified Center for Disease Control and Prevention growth charts [6]. We interviewed the students and their parents, and we asked the participants about their personal information, including age, sex, educational level, type of school, students’ weight during traditional study and during virtual study, and the students’ height. To assess the impact of the change from the traditional classroom setting to that of online classes on students’ body weight, we asked the following questions: what types of electronic devices do students use to do their schoolwork? How many hours do the students use electronic devices for schoolwork and for non-schoolwork during the weekdays and the weekend? It was measured based on whether students used the devices for less than or more than five hours on an average day.

Furthermore, we inquired about the number of times per week that the students usually ate the following food items while doing their schoolwork: potato crisps, groundnuts, fried foods such as chicken wings, chicken fingers, cheese, etc., sweets such as cookies, chocolate or candy bars, biscuits, ice cream, soft drinks and energy drinks, and fast food such as pizza and burgers.

In addition, the participants were questioned about their daily physical activity, namely the amount of time they spent exercising (minimum one hour per day). The above-mentioned questions were asked again during the virtual classes to assess the impact of the COVID-19 pandemic on the participant’s weight in this phase. A few outcomes were rejected as there were missing values for stature and weight, and a few BMI standard deviation values were outside the range. Informed verbal consent was obtained from both the parents and students.

Statistical analysis

The data were analyzed using SPSS version 25 (Armonk, NY: IBM Corp.). Quantitative data are presented as the range (minimum, maximum), mean, and standard deviation (SD). Quantitative data are presented as the number and percentage. An independent sample t-test and one-way ANOVA were used to compare the intergroup differences in the quantitative data of two independent groups and of more than two groups, respectively. The chi-square test was used to compare the intergroup differences in the qualitative data of different groups. P <0.05 was considered statistically significant.

## Results

Sociodemographic characteristics of the participants

This study aimed to examine the effect of virtual education during the COVID-19 pandemic on weight as compared with the traditional education among school-age students in the western region of Saudi Arabia. In this cross-sectional study, a total of 518 participants (age, range: six to 18 years) were included in the data analysis based on the number of clinic interviews. The sample size was calculated by using the Raosoft software package (Seattle, WA: Raosoft, Inc.) single proportion equation. Based on the assumption that the rate of burnout is 50% and an error margin of 5% at the 95% confidence level of the calculated sample. The participants (518) were carried out by systematic random selection with proportional allocation. They were included based on their age and education level after taking consent from the parents; we excluded the patient younger than six or older than 18 and that students for refusal parent and incomplete missing interview. We collected the study-related information in three phases, specified by a four-month interval - phase 1, the first term from September 2019 to January 2020 (pre-COVID-19); phase 2, the second term from January 2020 to May 2020; and phase 3, both terms from September 2020 to May 2021.

Participants were divided into three groups according to their educational stages - primary school (54.4%), intermediate school (33.2%), and secondary school (12.4%) (Table 1). More than half of the students were studying in governmental schools (55.2%) and the remainder were studying in private schools (44.8). Most of the participants were from the western region (90.9%), and the remainder (9.1%) were from other regions.

**Table 1:** Demographics of the study population

Demographics		Percentage
Gender	Male	49.4%
	Female	50.6%
Educational stage	Primary	54.4%
	Preparatory	33.2%
	Secondary	12.4%
School type	Government	55.2%
	Private	44.8%
District	Mecca	90.9%
	Other districts	9.1%

Obesity

The results showed that the virtual study modality affected the students’ BMI, which increased from a mean of 19.24 to 20.08, before and after the COVID-19 pandemic, respectively. Table 2 shows that before the COVID-19 pandemic, 26.1% of the study cohort engaged in physical activity for one hour per week and 28% never engaged in physical activity on the weekend; after the onset of and during the COVID-19 pandemic, the percentage of physical inactivity during weekdays and weekends increased by 39% and 37.5%, respectively.

**Table 2:** Relation between BMI before and during COVID-19 with physical activity and other student characteristics Body mass index is expressed as mean ± standard deviation, and the other parameters are expressed as number (%). *Chi-square test used to calculate p-values of gender, educational stage, and school type. COVID-19: coronavirus disease 2019

	Hours	Gender*			Educational stage*				School type*		
		Male	Female	p-Value	Secondary	Intermediate	Primary	p-Value	Private	Governmental	p-Value
Duration of physical activity at weekdays before COVID-19	Never	17.60%	23.70%	0.01	26.60%	19.20%	20.20%	0.78	21.10%	20.30%	0.15
	< 1	16.00%	22.50%		15.60%	20.30%	19.50%		17.70%	20.60%	
	1	23.40%	28.60%		29.70%	23.30%	27.00%		28.40%	24.10%	
	2	26.60%	16.00%		17.20%	23.80%	20.60%		23.70%	19.20%	
	3	10.50%	5.70%		7.80%	9.90%	7.10%		4.70%	10.80%	
	4	2.70%	1.90%		0.00%	1.70%	3.20%		1.70%	2.80%	
	5 or >	3.10%	1.50%		3.10%	1.70%	2.50%		2.60%	2.10%	
Duration of physical activity at weekend before COVID-19	Never	26.20%	29.80%	0.41	32.80%	31.40%	24.80%	0.36	28.40%	27.60%	0.37
	< 1	13.30%	17.60%		12.50%	13.40%	17.40%		16.40%	14.70%	
	1	19.90%	21.00%		23.40%	19.20%	20.60%		23.30%	18.20%	
	2	19.10%	14.50%		17.20%	17.40%	16.30%		15.90%	17.50%	
	3	8.60%	8.40%		6.30%	10.50%	7.80%		6.50%	10.10%	
	4	5.90%	3.40%		4.70%	5.20%	4.30%		3.00%	5.90%	
	5 or >	7.00%	5.30%		3.10%	2.90%	8.90%		6.50%	5.90%	
Duration of physical activity at weekdays during COVID-19	Never	37.50%	40.50%	0.001	43.80%	34.30%	40.80%	0.27	42.20%	36.40%	0.11
	< 1	18.80%	26.70%		14.10%	23.30%	24.50%		18.50%	26.20%	
	1	17.20%	20.20%		17.20%	23.80%	16.00%		20.70%	17.10%	
	2	13.70%	9.50%		9.40%	11.60%	12.10%		9.50%	13.30%	
	3	7.80%	1.50%		7.80%	4.70%	3.90%		5.20%	4.20%	
	4	2.70%	1.10%		4.70%	1.20%	1.80%		3.00%	1.00%	
	5 or >	2.30%	0.40%		3.10%	1.20%	1.10%		0.90%	1.70%	
Duration of physical activity at weekend during COVID-19	Never	35.50%	39.30%	0.11	45.30%	40.10%	34.00%	0.03	38.80%	36.40%	0.28
	< 1	14.80%	17.90%		17.20%	11.60%	19.10%		13.40%	18.90%	
	1	17.60%	21.40%		7.80%	21.50%	20.90%		20.30%	18.90%	
	2	13.70%	9.90%		12.50%	16.90%	8.50%		10.80%	12.60%	
	3	5.50%	5.00%		7.80%	4.10%	5.30%		4.70%	5.60%	
	4	4.70%	3.40%		4.70%	2.30%	5.00%		6.00%	2.40%	
	5 or >	8.20%	3.10%		4.70%	3.50%	7.10%		6.00%	5.20%	

Table 3 illustrates that the incidence of overweight and obesity during the COVID-19 pandemic was 16.6% in the study population; among these individuals, 4.6% (n=24) were obese and 12.0% (n=62) were overweight. Obesity was associated with a lack of physical activity and a significant increase (p<0.001) in the number of screen-time hours due to virtual study. The mean usage duration of electronic devices for education on weekdays for more than five hours per day was 20.64.

**Table 3:** BMI comparison before and during the COVID-19 pandemic COVID-19: coronavirus disease 2019

	Percentage		Gender			Educational stage				School type		
	Obese	Overweight	Male	Female	p-Value	Primary	Intermediate	Secondary	p-Value	Private	Governmental	p-Value
BMI before COVID-19	6.20%	8.10%	19.85±6.27	18.63±6.10	0.03	17.58±5.98	20.75±5.26	22.47±7.23	<0.001	19.74±6.70	18.83±5.76	0.1
BMI during COVID-19	4.60%	12.00%	20.66±6.73	19.51±55.41	0.03	18.52±5.34	21.52±5.93	23.09±7.68	<0.001	20.39±5.93	18.82±6.26	0.29

BMI and gender

The comparison of BMI values before and during the COVID-19 outbreak showed an increased BMI mean for both sexes. However, the increase of BMI in female participants was higher than that in male participants. Before the COVID-19 pandemic, female participants exercised for one hour during weekdays (28.6%) and did not exercise on the weekend (29.8%). During the COVID-19 pandemic, the percentage of those who did not exercise on weekdays and weekends increased to 40.5% and 39.3%, respectively. The association of physical activity levels with BMI was significantly high in female participants both before and after the COVID-19 pandemic (p≤0.001).

BMI and educational stage

In addition, our analysis showed that primary school students had the highest comparison with other educational stages, and there was a significant positive association between the increasing BMI and the educational stage (p<0.001). The results revealed that before the pandemic, 27% of the primary school students exercised for one hour during weekdays, and 24.8% of them did not exercise during weekends. After the beginning of the pandemic, the percentage of participants who did not exercise during weekdays and weekends increased by 40.8% and 34%, respectively. Furthermore, regarding the use of electronic devices and the educational stage after the beginning of the COVID-19 pandemic, primary school students used electronic devices for more than five hours per day during weekdays for education (46.1%); however, on weekends, these participants used the devices for gaming (69.0%). Data regarding the BMI and school type are shown in Table 3, and the analysis showed no significant association between BMI and school type before and during the COVID-19 pandemic. Moreover, the analysis showed that there was no significant association between physical activity and school type on weekdays and weekends before and during the COVID-19 pandemic.

Use of electronic devices

There was no significant association between gender and mobile phone use (p=0.14). However, mobile phones were the most frequently used devices (52.0%) among male students before the COVID-19 pandemic, with usage duration of five hours or more (23.8%) on video games (63.9% and 75.7% on weekdays and the weekend {p=0.01 and p=0.00}], respectively). Among female participants, tablets were the most frequently used device (50.4%), and there was a highly significant association between tablet use and female sex (p≤0.001). Moreover, during the COVID-19 pandemic, a highly significant association has been found between tablet use and the female sex (p=0.004). Nonetheless, the most popular device for virtual education has been the personal computer among both male (50.0%) and female (48.5%) participants, with the longest usage duration noted in female participants for education (61.1%) and other uses (34.7%). However, no statistical differences were noted among the sexes for the duration of usage of electronic devices for education (p=0.83) and other uses (p=0.16).

**Figure 1:**
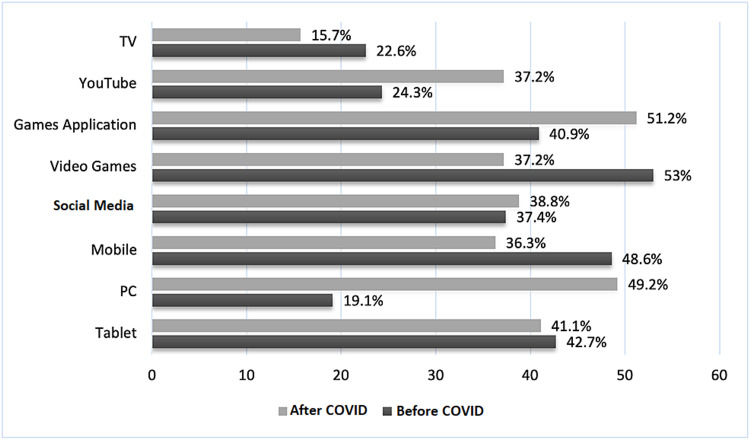
Usage of electronic devices during weekdays PC: personal computer; COVID: coronavirus disease

**Figure 2:**
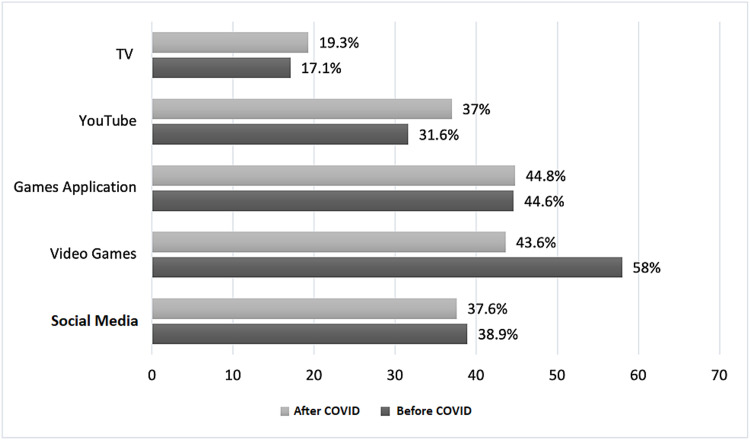
Usage of electronic devices during weekends COVID: coronavirus disease

Regarding electronic devices and school type, before the COVID-19 pandemic, tablets were used more often in private schools (54.7%), with a significant association (p≤0.001), whereas during the COVID-19 pandemic, personal computers (PCs) were used more often in private schools (59.1%), with a significant relationship (p≤0.001) (Figures 1, 2).

## Discussion

Worldwide, the COVID-19 pandemic has resulted in the enforcement of restrictions, including quarantine, school closures, and virtual modes of learning in different countries. Besides the cancelation of many sports activities, the above-mentioned restrictions led to reduced physical activity and an increase in the BMI of most students [5,7-12]. According to past research, the prevalence of obesity among Saudi Arabian has children increased [9,13]. In this study, the BMI of the students showed a significant change from normal weight to overweight with a prevalence of 12.0% for both genders from before to during the COVID-19 pandemic, whereas the prevalence of obese students decreased to 4.6%. In contrast, a Jordanian study found that the prevalence of both overweight and obese children increased to 24.1% during a lockdown, whereas the prevalence of overweight adolescents decreased to 20.7%, and the prevalence of obese adolescents increased to 16.4% compared to that before the COVID-19 lockdown [14]. The variance in the prevalence of the weights of the participants between the studies could be related to the differences and sensitivity of the methods used for data collection in each study.

This study found a significant association between gender and lack of exercise before and during the COVID-19 pandemic. In another study conducted in China by He et al., in 2020, both women and men with a BMI <24 gained weight, men with a BMI ≥24 lost weight, whereas women with a BMI ≥24 gained weight. During the semi-lockdown, the average amount of exercise declined significantly in both genders. Furthermore, during quarantine, body weight changes were inversely related to increases in physical activity [14]. Regarding electronic devices and gender Hancox and Poulton reported a strong relationship between BMI and television-watching in women [15]. However, the current study showed no differences between the two sexes.

In terms of BMI and educational stage, there was a significant association between an increase in BMI and the educational stage, and the study found that primary school students had the highest BMI compared to students in the other educational stages. On the other hand, a study in China among students has confirmed that the prevalence of overweight and obesity among high school and undergraduate students was significantly increased compared to that in other educational stages [16]. In the current study, the percentage of physical activity before the COVID-19 pandemic was higher in primary school students; thus, before the COVID-19 pandemic, 27% of participants exercised for one hour on weekdays, but during the COVID-19 pandemic, 40% and 37% of participants did not exercise on weekdays and weekends, respectively. Similarly, a study in the United States confirmed that during the pandemic, physical activity decreased, and children were in a sedentary lifestyle, leading to obesity and other diseases [5]. In addition, another study in Jordan showed that the percentage of children who did not exercise and play increased from 16.7% to 29.8% from before to after the pandemic lockdown, and they spent more than three hours daily on screen during the pandemic [17].

With regard to electronic devices and educational stage, this study found that during the COVID-19 pandemic, 46.1% and 69.0% of primary school students used electronic devices during weekdays for education and on weekends for gaming, respectively. In contrast, a study in the United States showed that girls and older children spent 90 minutes per day on electronic devices for homework and attended virtual lectures on both weekdays and weekends; during their leisure time, the children spent eight hours per day on other activities, such as playing video games and using the Internet [5].

Limitations

The main limitation of this study is its cross-sectional design which prevents reliable causal inferences. Further prospective studies are required to verify these findings. Other limitations include the small sample size of the study, limited coverage area, and the exclusion of certain results because they were incomplete. Nonetheless, this study included very recent data that reflects the effect of the COVID-19 pandemic on student lifestyles.

## Conclusions

In conclusion, the COVID-19 pandemic has caused significant disruptions to students' routines and lifestyles. This study showed that there is a multifactorial relationship between virtual education and students’ BMI. An increase in BMI (12%) was reported after the onset of the COVID-19 pandemic in overweight students, with significance for primary school and female students. In female students, there was a significant relationship between BMI and reduced physical status. However, there was no association between the school type and BMI. Students, specifically female students, who spent ≥5 hours per day on electronic devices had a higher BMI, specifically using tablets. The results of this study are similar to those of other studies that examined the impact of the COVID-19 pandemic on children's lifestyles. Therefore, the findings indicate the need for further research to avoid the negative consequences of overweight and obesity in school students due to many obesogenic factors as a result of restrictions during the COVID-19 pandemic.
